# Genetic variants involved in specialized DNA replication and their relation with breast cancer risk, disease progression and patient prognosis

**DOI:** 10.1186/1753-6561-7-S2-P7

**Published:** 2013-04-04

**Authors:** Ana PC Brandalize, Roberto Minozzo, Lavínia Schüller-Faccini, Jean-Sebastien Hoffman, Christophe Cazaux, Patricia Ashton-Prolla

**Affiliations:** 1 Department of Genetics, Federal University of Rio Grande do Sul, Porto Alegre, Brazil; 2 Experimental Research Center, Hospital de Clínicas de Porto Alegre, Porto Alegre, Brazil; 3 National Institute of Populational Genetics, INAGEMP, Porto Alegre, Brazil; 4 Cancer Research Center of Toulouse, INSERM U1037, University Paul Sabatier, University of Toulouse, Toulouse Oncopole, Toulouse, France

## Background

The molecular mechanisms involved in genetic instability, which is a driving force of cancer cells from earlier stages of pathologenesis, are not fully understood. Current evidence shows that overexpression of Pol θ, a “DNA repair” polymerase specialized in the replication of damaged DNA, which is altered in breast tumors, is not a passive agent in tumor development and is able to predict patient outcome. Aberrant *POLQ* expression may be related to genetic instability, and also resistance to “replicative stress”, leading to changes in replicating parameters and consequent tumor development. The objective of this project is to analyze genetic variants related to *POLQ* as new population biomarkers of risk, progression and prognosis in hereditary (HBC) and sporadic (SBC) breast cancer in Brazil.

## Materials and methods

Single Nucleotide Polymorphisms (SNPs) were systematically identified through the NCBI database. SNPs identified result in amino acid exchange in the protein and are located within exons or promoter regions. Most SNPs have not been tested in population-based studies. We recruited 211 breast cancer patients (94 SBC and 114 HBC) and 206 women without cancer. In this case-control study, we first genotyped seven SNPs (rs61757736, rs55748151, rs41545723, rs1381057, rs587553, rs13065220, rs3806614) using Taqman Real Time PCR. Data were analyzed using SPSS 18.0.

## Results

Interestingly, the rs581553SNP located in a promoter region was associated with HBC (g.121265913T>C; HBC TT=16, Control TT=8; OR=2.01, CI95%= 1.32-3.32; p<0.001). Although the Chi-Square analysis did not show any statistical difference between groups for the other SNPs, the HBC group showed more polymorphic genotypes than SBC and Control groups regarding the rs1381057 SNP (c.7538T>C; SBC TT=7, HBC TT=13, Control TT=8).

## Conclusions

These results suggest that *POLQ* germline variation may be related to cancer progression in this patient group. Additional SNPs are being analyzed and the correlation between genotype and relevant clinical variables for breast cancer prognosis will be evaluated.

## Financial support

CNPq, FIPE-HCPA, INAGEMP.

